# Characteristics of Bacterial Microbiota in Different Intestinal Segments of Aohan Fine-Wool Sheep

**DOI:** 10.3389/fmicb.2022.874536

**Published:** 2022-04-28

**Authors:** Yuhao Ma, Xiaotian Deng, Xue Yang, Jiankui Wang, Tun Li, Guoying Hua, Deping Han, Lai Da, Rui Li, Weiheng Rong, Xuemei Deng

**Affiliations:** ^1^Key Laboratory of Animal Genetics, Breeding, and Reproduction of the Ministry of Agriculture and Beijing Key Laboratory of Animal Genetic Improvement, China Agricultural University, Beijing, China; ^2^Inner Mongolia Academy of Agriculture and Animal Husbandry, Hohhot, China; ^3^Inner Mongolia Grassland Jinfeng Animal Husbandry Co., Ltd., Chifeng, China

**Keywords:** sheep, microbial diversity, intestinal segments, stress tolerance, high-throughput sequencing

## Abstract

The microbial community performs vital functions in the intestinal system of animals. Modulation of the gut microbiota structure can indirectly or directly affect gut health and host metabolism. Aohan fine-wool sheep grow in semi-desert grasslands in China and show excellent stress tolerance. In this study, we amplified 16S rRNA gene to investigate the dynamic distribution and adaptability of the gut microbiome in the duodenum, jejunum, ileum, cecum, colon, and rectum of seven Aohan fine-wool sheep at 12 months. The results showed that the microbial composition and diversity of the ileum and the large intestine (collectively termed the hindgut) were close together, and the genetic distance and functional projections between them were similar. Meanwhile, the diversity index results revealed that the bacterial richness and diversity of the hindgut were significantly higher than those of the foregut. We found that from the foregut to the hindgut, the dominant bacteria changed from Proteobacteria to Bacteroidetes. In LEfSe analysis, *Succiniclasticum* was found to be significantly abundant bacteria in the foregut and was involved in succinic acid metabolism. *Ruminococcaceae* and *Caldicoprobacteraceae* were significantly abundant in hindgut, which can degrade cellulose polysaccharides in the large intestine and produce beneficial metabolites. Moreover, *Coriobacteriaceae* and *Eggthellaceae* are involved in flavonoid metabolism and polyphenol production. Interestingly, these unique bacteria have not been reported in Mongolian sheep or other sheep breeds. Collectively, the gut microbiota of Aohan fine-wool sheep is one of the keys to adapting to the semi-desert grassland environment. Our results provide new insights into the role of gut microbiota in improving stress tolerance and gut health in sheep.

## Introduction

Every part of an individual animal has a microbial community (Neish, 2009). The microbial community exists in a symbiotic relationship with the host (Ley et al., 2006a). This complex collection of microorganisms is called the microbiota, and their genetic material is called the microbiome (Turnbaugh et al., 2007). Animal intestinal microbes are dynamic; during the host’s life cycle, the microflora undergo significant changes due to factors, such as diet, environment, and disease state, among others (Shreiner et al., 2015). Microbes contribute to energy homeostasis, metabolism, intestinal epithelial health, immune activity, and nerve development in animals (Cho and Blaser, 2012). In addition, intestinal microorganisms play an important role in the development of animals. The addition of corn bran to the diet of weaned piglets induced changes in the ratio of Firmicutes to Bacteroidetes changing the microbial diversity and enhancing the anti-inflammatory response of the organism (Liu et al., 2018). Similarly, the addition of caragana to sheep diets changes the composition of intestinal microbes in the body, improving the meat quality of sheep in terms of tenderness and fatty acid content (Zhang et al., 2021).

At present, it is generally considered that the distribution of intestinal bacteria in different intestinal segments is different, and is related to the function of the particular intestinal segment. In a study on broiler chickens, Firmicutes was the dominant genus in the intestinal tract, and Bacteroidetes, as an important type of phylum, only occupies 50% of the abundance in the cecum (Xiao et al., 2017). A study on pigs revealed that Proteobacteria was the dominant phyla in the small intestine, while Firmicutes was the dominant phylum in the large intestine, accounting for 80% of the microbial population, followed by Proteobacteria (Zhao et al., 2015). In Mongolian sheep growing in the Gansu province of China, the proportion of Firmicutes and Bacteroidetes among the intestinal microbes reached 80%, and there was no consistent bacterial ratio among intestinal segments (Zeng et al., 2017). However, the intestinal microbial composition of a camel’s ileum, cecum, and colon was relatively similar, and Firmicutes and Bacteroidetes accounted for 50–60% of the intestinal microbiota (He et al., 2018). This shows that there are obvious differences in intestinal microbes between monogastric animals and ruminants. Meanwhile, different types of ruminants have different compositions of intestinal microbes. These differences are caused by the characteristics of the animal and its natural environment (Guo et al., 2020b). In our analysis, we considered the duodenum and jejunum as the foregut, and the ileum and large intestine as the hindgut based on the microbial composition.

The current analysis of sheep intestinal microbes focuses on the exploration of fecal microbes and microbes in individual intestinal segments. Some studies have reported that the fecal microbiota cannot represent the microbial composition of intestinal segments (Zhao et al., 2015; Donaldson et al., 2016). Moreover, for the study of a single intestinal segment, the correlation between the intestinal segments is usually lacking, and only the characteristics of a single intestinal segment can be displayed (Al-Masaudi et al., 2017; Zhang et al., 2018; Lin et al., 2021). To study the composition of the gut microbiome of Aohan fine-wool sheep, we determined the complete microbiome spectrum of the adult sheep gut. We also systematically studied the changes in the intestinal microbiota between the foregut and hindgut to explore the stability of microorganisms in different intestinal segments.

Aohan fine-wool sheep is produced in Northeast China. It is a wool-meat dual-purpose breed developed by crossing Chinese Mongolian sheep as female parent and Soviet Caucasian sheep and Gustav sheep as male parent. Aohan fine-wool sheep have the advantages of roughage tolerance and high adaptability (Xiao-ping, 2009; Cui et al., 2014). Aohan sheep farms are located in regions with low annual rainfall, low winter temperatures, and strong winds accompanied by sandstorms and dust problems. Therefore, the quantity and quality of pastures in Aohan sheep farms are poor (Masters et al., 1990). Aohan fine-wool sheep have been selected and bred for generations, becoming an excellent breed that can be grown in arid desert areas, for wool and meat. In this study, we analyzed the overall gut microbial composition and differential bacterial genera in Aohan fine-wool sheep and compared the differences in microbial composition between the foregut and hindgut. In addition, we performed the functional analysis of microorganisms and analyzed the importance of intestinal microorganisms in influencing breed characteristics. The results showed the relationship between intestinal microorganisms of Aohan fine-wool sheep and their breed characteristics and environmental adaptability.

## Materials and Methods

### Ethics Statement

All experimental designs and operations were approved by the Animal Care and Use Committee of China Agricultural University and performed in accordance with the “Guidelines for Experimental Animals” of the Ministry of Science and Technology, Beijing, China (Permit number: SKLAB-2012- 04-07).

### Animals and Sample Collection

**FIGURE 1:**
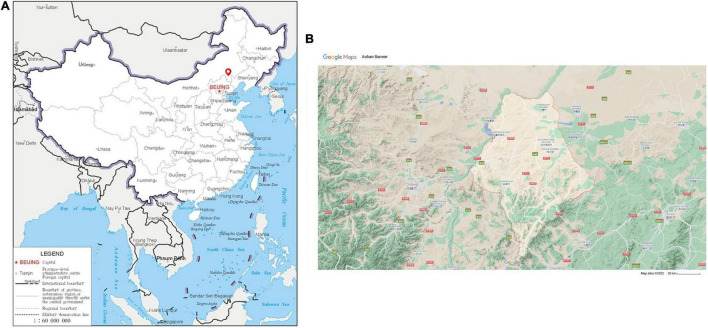
The sampling site of this study was Aohan Banner. **(A)** The geographic location of raising Aohan fine-wool sheep is indicated by red coordinates. Map from the China Department of Natural Resources. Approval number: GS(2019)1652. **(B)** The red marks indicate the geographical areas where the Aohan fine-wool sheep live; these areas have a semi-desertified grassland environment. The scale bar is 20 km, source: Google Maps.

During the research, seven Aohan fine wool rams were obtained from JinFeng Animal Husbandry Co., Ltd. (Chifeng City, Inner Mongolia Autonomous Region, China) (42°15′28.1″N, 118°53′12.7″E) (Figure 1A). All sheep were raised according to standard livestock management methods, including the same temperature and humidity (The annual average temperature is 4.9–7.4°C, and the annual precipitation is 218–595 mm), the same feed (Lambs are fed with the milk of ewes, and as adults, the feed is mainly maize straw and alfalfa hay), the same grazing time, and sufficient drinking water (Figure 1B). At the age of 12 months, seven Aohan fine wool sheep were euthanized and slaughtered. Fresh intestinal contents (∼10 g) were collected from duodenum, jejunum, ileum, cecum, colon, and rectum. Samples were placed in liquid nitrogen immediately after collection, and then transferred to –80°C ultra-low temperature freezer for storage.

### DNA Extraction, PCR Amplification, and Illumina MiSeq Sequencing

Microbial genome was extracted according to experimental steps using TIAN amp Genomic DNA Kit (TIANGEN Bio-Tek Co., Ltd., Beijing, China). The quality of DNA was detected by 0.8% agarose gel electrophoresis, and DNA was quantified by UV spectrophotometer. V3-V4 region of 16S rRNA gene was amplified using the following primers: ACTCCTACGGGAGGCAGCA (forward) and GGACTACHVGGGTWTCTAAT (reverse). NEB’s Q5 high-fidelity DNA polymerase (NEB Bio-Tek Inc., Ipswich, MA, United States) was used for amplification, the system is 25 μl: 5× Q5 Reaction Buffer 5 μl, 10 mM dNTPs 0.5 μl, 10 μM Forward and Reverse Primer 1.25 μl for each, Template DNA 1 μl, ddH_2_O 16 μl. The thermal cycle includes initial denaturation at 98°C for 2 min, denaturation at 98°C for 15 s, annealing at 55°C for 30 s, extension at 72°C for 30 s, and final extension at 72°C for 5 min, holding at 4°C, and cycling 30 times. AXYGEN gel recovery kit (Corning Bio-Tek Inc., NY, United States) was used for gel cutting and recovery, and fluorescence quantification of PCR amplification and recovery products was performed. The fluorescence reagent is Quant-iT PicoGreen dsDNA Assay Kit (Thermo Fisher Scientific Inc., West Palm Beach, FL, United States), and the quantitative instrument is FLx800 Microplate reader (Agilent BioTek Inc., Santa Clara, CA, United States). After fluorescence quantification, the samples were mixed proportionally according to the sequencing requirements of each sample. The sequencing library was prepared using TruSeq Nano DNA LT Library Prep Kit (Illumina BioTek Inc., San Diego, CA, United States). The constructed libraries, inspected for quality using Agilent High Sensitivity DNA Kit (Agilent BioTek Inc.), presented only a single peak and no linker. MiSeq sequencer (Illumina BioTek Inc.) was used to carry out 2 × 300 bp paired-end sequencing, and the corresponding reagent is MiSeq Reagent Kit V3 (600 cycles) (Illumina BioTek Inc.). In order to ensure quality, the insert range for sequencing was 200–450 bp.

### Bioinformatics Analysis and Statistics

The raw data goes through the steps of remove primer, quality filtering, denoise, splicing and de-chimerism to obtain clean reads. This process is realized through the QIIME2 DADA2 platform (Callahan et al., 2016; Bolyen et al., 2018, p. 2). Each deduplicated sequence generated using DADA2 quality control is called an operational taxonomic unit (OTU) representative sequence. By performing statistics on the leveled OTU table, the specific composition table of the microbial communities in each sample at each classification level can be obtained. Through this table, calculate the number of classification units contained in different samples at each classification level. In order to analyze the indicators of microbial richness, diversity and evenness, we conducted an Alpha diversity analysis. Next, we used bray_curtis distance algorithm to performed principal coordinate analysis (PCoA) and cluster dendrogram, and the unweighted pair-group method was used to calculate arithmetic mean. Linear discriminant analysis effect size (LEfSe) analysis realizes simultaneous differential analysis of all classification levels of microorganisms (Segata et al., 2011). Meanwhile, it searches for robust different species between groups.

PICRUSt2 is a software that predicts the functional abundance of samples based on the abundance of marker gene sequences in the samples (Douglas et al., 2020). We used the MetaCyc functional database^1^ to predict 16S rRNA gene sequences. And based on the data results, we obtained different metabolic pathways between different subgroups.

GraphPad Prism (version 8.0) was used for statistical analysis. The Repeated Measures ANOVA test method was used to detect the differences of microbial abundance between intestinal segments. *F* > 1 indicated that difference of the mean squares between groups and within the groups is statistically significant. The criterion of significance was conducted at *P* < 0.05.

## Results

### Sequencing Results and Bacterial Diversity of Different Intestinal Segments

**FIGURE 2:**
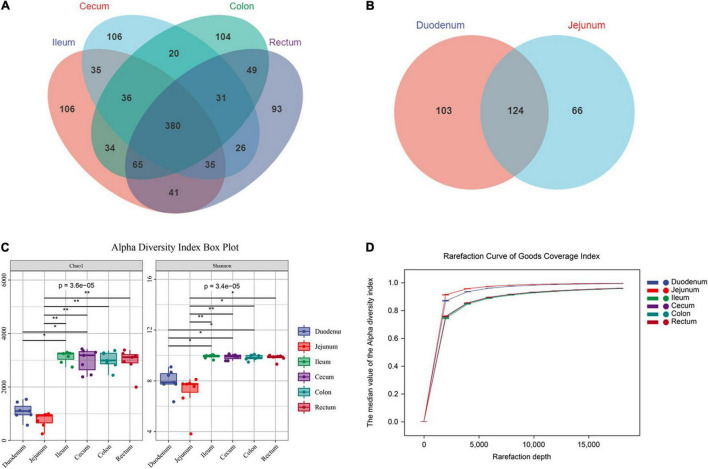
Sequencing results and statistical analysis of diversity. **(A)** Venn diagram showing the OTUs shared among the hindgut segments. **(B)** Venn diagram showing the OTUs shared between the foregut segments. **(C)** The Chao1 and Shannon indices of six intestinal segments. Significantly different indices were tested by Kruskal-Wallis test with adjusted *P value of < 0.05, **P > 0.01. **(D)** Rarefaction curve of Good’s coverage index. Each curve represents the mean within the group.

In total, we obtained 2,455,588 raw reads from 6 intestinal segments of seven sheep. After filtering out, denoising, chimera checking, and singleton checking, we obtained clean reads of 1,302,080 sequences. And 17,379 operational taxonomic units (OTUs) were obtained by clustering at 97% identity (Supplementary Materials 1, 2). To study the commonality and peculiarity of the microbes, we used a Venn diagram to calculate the number of OTUs shared by different intestinal segments between the groups. The frequency of the screening samples was 50%. First, we analyzed the intestinal segments of the hindgut. The number of core OTUs shared by the four hindgut segments was 380 (Figure 2A). The number of OTUs observed in the foregut was 124, which was less than that observed in the hindgut (Figure 2B). The results showed that the microbial abundance of the foregut was lower than that of the hindgut.

To prove the accuracy of the analysis results, we conducted a diversity analysis of intestinal microbes and used Chao1 and Shannon indexes to analysis. The results showed that the diversity and richness of intestinal microbes were higher in the hindgut (*P* < 0.01, *F* > 1.00), and there was no significant difference in microflora among the hindgut segments (ileum, cecum, colon and rectum; *P* > 0.05, *F* = 0.78; Figure 2C). The Good’s coverage index of each sample was more than 90%, and the curve tended to be flat. Among them, the index of the foregut was steep, indicating that the abundance of the detected OTUs was higher in the foregut. Meanwhile, the results show that the sequencing data were sufficient to cover all the bacterial communities (Figure 2D).

### Cluster Analysis and Microbial Composition Among Different Intestinal Segments

**FIGURE 3:**
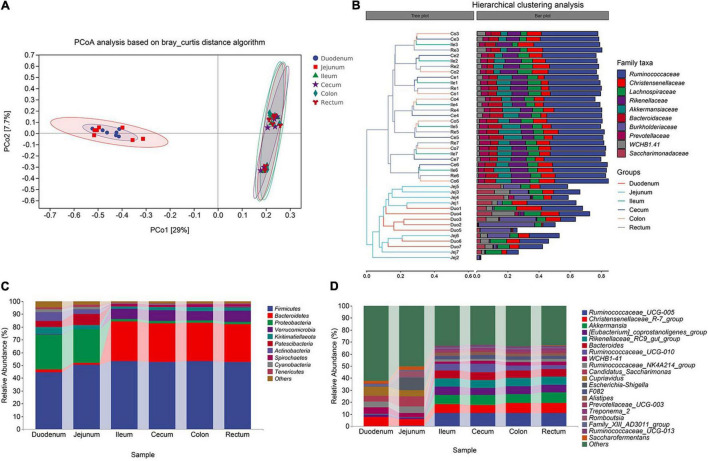
Cluster analysis of Aohan fine-wool Sheep. **(A)** Principal coordinate analysis (PCoA) based on all samples. **(B)** The hierarchical tree shows the UPGMA clustering result. The abscissa indicates the distance between samples, the number after the group abbreviation represents the individual number, and the branch length indicates similarity. On the right is the stacked histogram of the top 10 abundant bacterial families in the sheep intestine. The abscissa indicates the proportion of bacteria. **(C)** The phylum-level microbial composition of each intestinal segment. **(D)** The genus-level microbial composition of each intestinal segment.

In previous studies on the cluster analysis of intestinal microbes in sheep, the correlation between the ileum and other intestinal segments was not accurately defined (Zeng et al., 2015; Wang et al., 2017). Therefore, we assumed that the bacterial compositions of the ileum and large intestine were similar. To confirm this hypothesis, we analyzed the microbial community composition of the six intestinal segments. And found that the microbial composition of the foregut and the hindgut was very different, and that of the intestinal segment ileum and the large intestine was similar. In addition, the degree of correlation in the microflora of the duodenum and jejunum in the foregut was not too high, with 29.0 and 7.7% variations explained by principal component 1 (PC1) and PC2, respectively (Figure 3A). Next, we conducted ANOSIM similarity analysis, and the results showed that the intestinal microbial structures of the foregut and hindgut were significantly different (*P* < 0.01, *R* = 0.54) (Supplementary Material 3).

In the hindgut, for the similar microbial composition clusters, the similarity between individuals in the same intestinal segment is higher than that between different intestinal segment within individuals (Figure 3B). The microorganisms in the hindgut mainly included *Ruminococcaceae*, *Christensenellaceae*, *Lachnospiraceae*, *Rikenellaceae*, and *Akkermansiaceae*. These five types of bacteria accounted for 65% of all the gut microbes (Figure 3B). The similarity in microbial composition may be related to functional consistency. In the foregut, the duodenum and jejunum were not clustered among individuals, and the jejunum was quite different among individuals. *Burkholderiaceae*, *Ruminococcaceae*, and *Saccharimonadaceae* were the dominant bacterial families. To judge whether the degree of dispersion of samples within a group is different among different groups, we used the ADONIS permutation test. The degree of dispersion of the foregut was significantly higher than that of the hindgut, and there was no significant difference in the foregut group (Supplementary Material 4).

In the analysis of microbial composition, we focused on exploring the top 10 bacteria at the phylum and genus levels. We analyzed the composition of microorganisms at each taxonomic level. At the phylum level, Firmicutes (average 52.88%) and Bacteroidetes (average 30.23%) were the dominant bacteria in the hindgut. In the foregut, Proteobacteria (average 31.70%) and Firmicutes (average 47.44%) were the dominant bacterial phyla. The proportion of *Bacteroidetes* (hindgut 30.16%, foregut 1.95%), *Verrucomicrobia* (hindgut 8.02%, foregut 0.19%), and *Spirochaetes* (hindgut 2.08%, foregut 0.01%) in the foregut and hindgut was significantly different (*P* < 0.01, *F* > 1.00) (Figure 3C). This shows that these bacteria are more active in the hindgut, indicating their role in the fermentation and water absorption processes of the hindgut. At the genus level, *Ruminococcaceae_UCG-005*, *Christensenellaceae_R-7_group*, *Akkermansia* (sum 26.14%) were the dominant genera in the hindgut. The proportion of *Christensenellaceae_R-7_group* (hindgut 7.43%, foregut 7.06%) remained the same throughout the intestine (*P* > 0.50, *F* > 1.00) (Figure 3D). Compared to the foregut, most of the annotated bacteria were more abundant in the hindgut. Additionally, *Candidatus-Saccharimonas* was a unique bacterium in the foregut.

### Microbial Communities of Different Intestinal Segments

**FIGURE 4:**
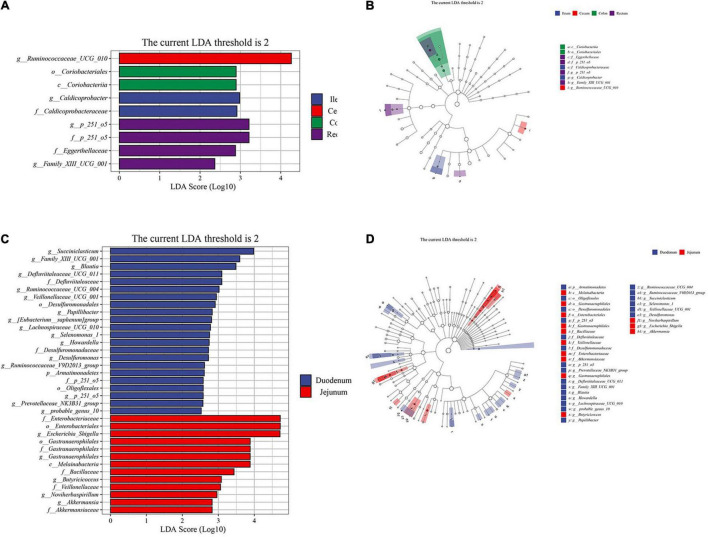
Linear discriminant analysis (LDA) effect size (LEfSe) analysis of the intestinal segments of Aohan fine-wool sheep. The LEfSe analysis histogram of hindgut **(A,C)** foregut. The ordinate is the taxa with significant differences between groups, and the abscissa is a bar graph displaying the LDA logarithmic score value of each taxon. The longer the length, the more significant the difference of the taxon, and the color of the bar graph indicates the sample group with the highest abundance corresponding to the taxon. The LEfSe analysis branch diagram of hindgut **(B,D)** foregut. The node size corresponds to the average relative abundance of the taxa, and the hollow nodes represent taxa with insignificant differences between groups. The letters identify the names of taxa that differ significantly between the groups.

To further explore the differences between the samples, we conducted a Kaplan-Meier analysis. According to the results of the cluster analysis, we performed the Wilcoxon test and LEfSe analysis on the foregut and hindgut, respectively. (Supplementary Material 5). The genus of more abundant bacteria in the cecum was *Ruminococcaceae_UCG_010*, and *Caldicoprobacter* in the ileum was a significantly abundant bacteria belonging to the Firmicutes phylum (LDA > 2, *P* < 0.05; Figure 4A). *Coriobacteriales* in the colon belongs to the phylum Actinobacteria. The rectum included significantly abundant bacteria *Family_XIII_UCG_001* under *Clostridium*, *Eggerthellaceae* under *Actinomycetes*, and *p_251_o5* under *Bacteroides* (LDA > 2, *P* < 0.05; Figure 4B). In the analysis of the foregut, we found that the significantly abundant bacteria of the duodenum mainly included *Succiniclasticum* under *Acidaminococcaceae*. The abundance of *Ruminococcaceae*, and *Defluviitaleaceae* under the class of *Clostridia* was also significantly higher than that in the jejunum (LDA > 2, *P* < 0.05; Figure 4C). *Escherichia_Shigella*, *Akkermansiaceae*, *Veillonellaceae*, and *Butyricicoccus* were more abundant in the jejunum compared to duodenum, which may be related to the specific digestive function of the jejunum (LDA > 2, *P* < 0.05; Figure 4D).

### Microbial Function Prediction and Intestinal Metabolic Pathways

**FIGURE 5:**
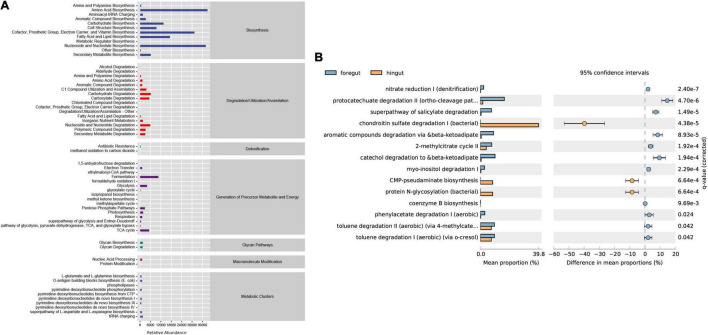
Predictive analysis and statistics of foregut and hindgut microbial function. **(A)** The abundance of the differential metabolic pathways based on the MetaCyc database. The abscissa is the abundance count of the classification, the ordinate is the functional pathway of MetaCyc’s second classification level, and the rightmost is the first-level classification to which this pathway belongs. **(B)** The differential analysis of metabolic pathways based on the metagenomeSeq method. Light blue represents the foregut and light yellow represents the hindgut. The right ordinate is the corrected *q*-value and the left ordinates are different pathway labels.

To investigate the functional differences in the intestinal microbiota of Aohan fine-wool sheep, we performed a functional analysis of microbiota using PICRUST2 (Supplementary Material 6). First, we counted the abundance of metabolic pathways in six intestinal segments using the MetaCyc database as a reference. The abundance statistics of functions revealed that more microbial functions were related to biosynthesis and metabolism (Figure 5A). The abundance of the biosynthesis processes was significantly higher than that of the other metabolic pathways. The main biological pathways in the intestine included amino acid biosynthesis, nucleoside and nucleotide biosynthesis, biosynthesis of cofactors, prosthetic groups, electron carriers, and vitamins, and the biological processes of biosynthesis, and fatty acid and lipid biosynthesis.

To investigate the differences between the intestinal metabolic pathways, we used *q*-value < 0.05 as the standard for the differential enrichment analysis. The results showed that the biological pathways of catechol degradation, salicylic acid degradation, aromatic compound degradation, and the citric acid cycle pathways are more active in the foregut. In contrast, the hindgut mainly includes the biological processes of chondroitin sulfate degradation and protein N-glycosylation synthesis (Figure 5B).

## Discussion

This study conducted a comprehensive analysis of the intestinal microbes of Aohan fine-wool sheep. The 16S rRNA gene sequencing method was used to analyze the microbial structure, composition, significantly abundant bacteria, and potential functions of each intestinal segment.

The small intestine is an important organ for digestion and absorption. The large intestine mainly absorbs water and absorbable nutrients. It can also produce acetate, propionate, and butyrate as sources of nutrients for intestinal cells (Miller and Wolin, 1996). The results of the PCoA analysis revealed that the foregut was significantly separated of hindgut. Our results are consistent with the analysis results in Mongolian sheep (Zeng et al., 2017). Our study showed that the intestinal microbial diversity and richness of the foregut of Aohan fine-wool sheep were significantly lower than those of the hindgut. Previous studies have confirmed that the microbial diversity in the small intestine is low (Eckburg et al., 2005). The microbial population in the small intestine is usually affected by a host of adverse factors, such as low pH, faster transit time, and exposure to bile acids and antimicrobial peptides. Studies have shown that Firmicutes and Proteobacteria, which dominate the small intestine, are more tolerant of these factors (Angelakis et al., 2015; Leone et al., 2015). This explains the abundance of Proteobacteria in the foregut of Aohan fine-wool sheep. Proteobacteria are also the dominant phylum on other species, including rumen of calves (Rey et al., 2014) and Yimeng black goats (Li et al., 2021). Interestingly, in previous studies on Mongolian sheep (raised in the Gansu province of China), Proteobacteria was not found to be the dominant phylum (Zeng et al., 2017). However, in camels (bred in Xilin Gol, Inner Mongolia Autonomous Region, China), the results were similar to those from our analysis (He et al., 2018). This indicates that the intestinal microflora is influenced by differences in rearing environments. The Bacteroidetes phylum is the dominant phylum in the large intestine, and the same results have been found in other mammals (Donaldson et al., 2016). The large intestine is characterized by a slow flow rate and a neutral to slightly acidic pH. It is beneficial for the colonization of Bacteroidetes (Flint et al., 2012). In general, our results indicate that in Aohan fine-wool sheep, the intestinal environment behind the ileum is more uniformly characterized by mild pH and low-speed transport. At the phylum level, the dominant phylum of gut microbes in ruminants and monogastric animals were consistent.

The results of our study indicated that the same microbial population exists in the foregut and hindgut and performs functions in the gut. Firmicutes and Bacteroidetes were the dominant flora in the hindgut. It participates in energy metabolism and affects obesity (Ley et al., 2006b; Komaroff, 2017). In addition, we found that the abundance of *Christensenellaceae* and *Lachnospiraceae* in the foregut and hindgut were relatively stable (*P* > 0.05), indicating that they play an important role in the intestinal tract. The *Christensenellaceae_R-7_group* is a member of the *Christensenellaceae* family. The *Christensenellaceae* family is a relatively new bacterial family that has previously been related to the host’s health (Waters and Ley, 2019). Moreover, *Christensenellaceae* is positively correlated with protein catabolism and intestinal metabolites of dietary animal proteins (Beaumont et al., 2017; Manor et al., 2018). In a study on ruminants, *Christensenellaceae_R-7_group* improved the development of the rumen and increased the absorption and digestion of nutrients (Couch et al., 2020; Huang et al., 2021). These results indicate that *Christensenellaceae* may be an important part of the gastrointestinal tract of ruminants. *Lachnospiraceae* is the main component of the intestinal microbiota of ruminants (Kittelmann et al., 2013), and is closely related to the production of butyrate (Haas and Blanchard, 2017). Our results are consistent with those of previous studies (Freetly et al., 2020; Li et al., 2020) and indicate that these stable bacteria are involved in the growth of ruminants.

In this study, we found significantly abundant bacteria in each intestinal segment. Interestingly, previous reports on the gut bacterial of Mongolian sheep were not focused on these bacteria, possibly due to differences in feeding environment and breed adaptation (Zeng et al., 2015, 2017). *Butyricicoccus* is a type of bacteria that produces butyric acid, providing butyrate as the main nutrient for intestinal epithelial cells (Geirnaert et al., 2014). *Akkermansiaceae* is related to gastrointestinal homeostasis and metabolic balance (Clarke et al., 2014). These foregut bacteria may be responsible for imparting the characteristics of strong adaptability and tolerance to rough feeding in Aohan fine-wool sheep. The significantly abundant bacteria in the hindgut mainly included *Caldicoprobacteraceae* and *Ruminococcaceae*. These bacteria can ferment a variety of nutrients and produce volatile fatty acids. In addition, indigestible polysaccharides can be used to produce metabolites that are beneficial to the intestinal tract (Deng et al., 2017; Guo et al., 2020a). A study showed that *Ruminococcaceae-UCG-010* and *UCG-005* (at the genus level) are related to the degradation of starch and fiber in ruminants (Kim et al., 2011). These communities may contribute to further fermentation of feed in the cecum. However, these core bacteria were not highly abundant in the cecum of Small Tail Han sheep (Zhang et al., 2018). This difference may be related to the characteristics of the breed and the distribution area. Small Tail Han sheep are distributed in the Shandong Province of China and are known for their very high rates of reproduction and extremely high fecundity. However, the meat quality of Small Tail Han sheep is worse than that of Mongolian sheep (Cannas, 2011). Compared with Mongolian sheep, the bacteria identified in this study has not been reported before (Zeng et al., 2017). However, in other ruminants, such as sika deer, *Ruminococcaceae-UCG-010* is reported to be the main bacterial species in the hindgut, where it degrades cellulose and produces short-chain fatty acids (SCFAs) (Li et al., 2019). Similar results have been reported in newborn calves (Elolimy et al., 2020). These observations are consistent with the results of our study. *Coriobacteriaceae* and *Eggerthellaceae* belong to the phylum Actinobacteria. Studies have indicated that the abundance of *Eggerthellaceae* is positively correlated with feed efficiency (Bach et al., 2019). Previously, research on the function of these highly abundant bacteria revealed that strains of *Coriobacteriaceae* and *Eggerthellaceae* are particularly involved in the metabolism of daidzein and genistein, and they can convert to food polyphenols (Soukup et al., 2021). However, The *Coriobacteriaceae* and *Eggerthellaceae* have not been observed and analyzed in other breeds of fine-wool sheep (Yang et al., 2021). In this study, the ability of Aohan fine-wool sheep to metabolize fiber polysaccharides and flavonoids may be related to the grazing environment. The grassland composition of semi-desert pastures is complex. In the current study, these bacteria found in the hindgut were not of interest in other breeds of sheep. Increased concentrations of these bacteria, such as *Caldicoprobacteraceae* and *Ruminococcaceae* in the hindgut, enhance the digestibility of crude fiber while producing beneficial metabolites.

The microbial potential function analysis revealed that the foregut plays an important role in the metabolism of biomass and produces important biosynthetic precursors. The methyl citrate cycling pathway in the foregut can metabolize propionate to pyruvate (Brock et al., 2002). Meanwhile, the metabolism of catechol and the degradation of salicylic acid is more active in the foregut. This pathway produces the energy substrates succinate and acetyl CoA, which are involved in energy metabolism (MacLean et al., 2006; Cámara et al., 2007). *Succiniclasticum* seems to be involved in the metabolite transformation process, which provides energy to ruminants during the conversion of succinic acid to propionic acid (Van Gylswyk, 1995). Therefore, the combined action of these substances and bacteria provides ruminants with additional energy. Our analysis showed that the metabolism of the hindgut involves the degradation of polysaccharides and the biosynthesis of carbohydrates (Olson, 2002). However, PICRUSt2 still has drawbacks in predicting the potential functions. Since 16S rRNA gene amplification is based on sequencing and analysis of hypervariable regions, functional prediction cannot provide resolution to distinguish strain-specific functionality, this leads to an important limitation of PICRUSt2 and any amplicon-based analysis (Douglas et al., 2020). These results demonstrated the bacteria involvement in the process of metabolic conversion and predicted the potential relationship with the roughing tolerance of Aohan Fine Wool Sheep.

## Conclusion

This study revealed the unique gut microbial compositions of Aohan fine-wool sheep growing in a semi-desert environment. The structure and composition of gut microbes in Aohan fine-wool sheep are associated with stronger environmental adaptation and gut health. Our results revealed a robust energy metabolism in the foregut. The hindgut has a strong ability to digest crude fiber and produce SCFAs. Therefore, these findings provide a baseline for understanding the complex intestinal microbiota adapt to the living environment and provides new insights into improving stress resistance and gut health in sheep through microbes.

## Data Availability Statement

The datasets presented in this study can be found in online repositories. The names of the repository/repositories and accession number(s) can be found in the article/Supplementary Material.

## Ethics Statement

The animal study was reviewed and approved by the Animal Care and Use Committee of China Agricultural University. Written informed consent was obtained from the owners for the participation of their animals in this study.

## Author Contributions

XuD: experiment conception and ranch work arrangement. YM: ranch sampling, complete analysis, write article. XiD: complete the preliminary analysis of the article, ranch sampling. JW, XY, and TL: mainly involve in the sampling of pastures. DH participate in pasture sampling and provide assistance in experimental sample processing. LD, RL, and WR: provide laboratory animals and help with sampling. All authors have read and agreed to the published version of the manuscript.

## Conflict of Interest

RL was employed by Inner Mongolia Grassland Jinfeng Animal Husbandry Co., Ltd. The remaining authors declare that the research was conducted in the absence of any commercial or financial relationships that could be construed as a potential conflict of interest.

## Publisher’s Note

All claims expressed in this article are solely those of the authors and do not necessarily represent those of their affiliated organizations, or those of the publisher, the editors and the reviewers. Any product that may be evaluated in this article, or claim that may be made by its manufacturer, is not guaranteed or endorsed by the publisher.

## Footnotes

1
https://metacyc.org/
