# Supplementary figures and images for: Characteristics of Bacterial Microbiota in Different Intestinal Segments of Aohan Fine-Wool Sheep
Source: Front Microbiol. 2022 Apr 28;13:874536. doi: 10.3389/fmicb.2022.874536 (PMC9097873; doi:10.3389/fmicb.2022.874536)

R = 1 , P = 0.002

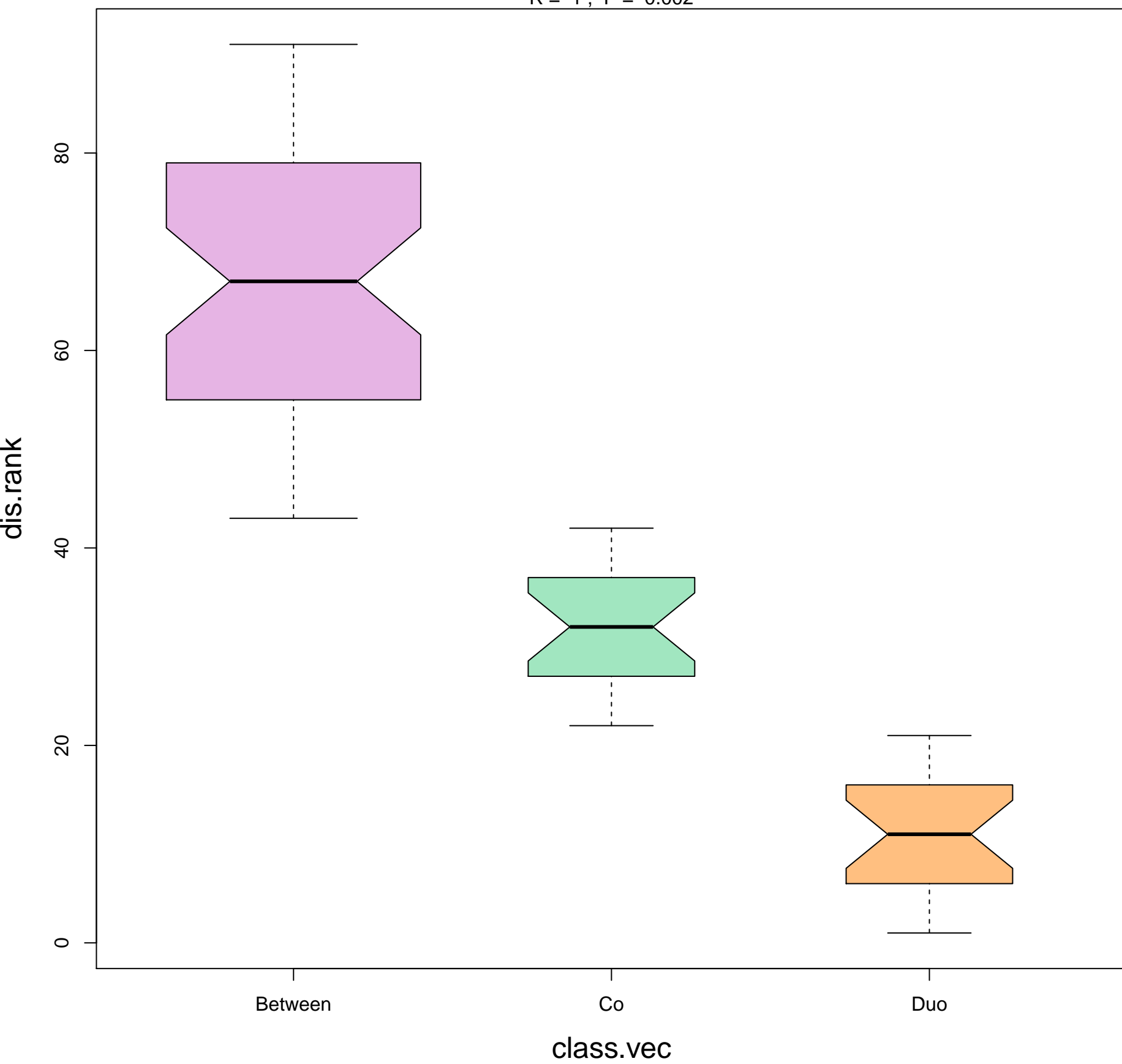

Supplement: Supplementary file 5 [file Data_Sheet_3.zip › Supplementary files 3 ANOSIM similarity analysis results/anosim.Co_Duo.pdf]

R = -0.014 , P = 0.452

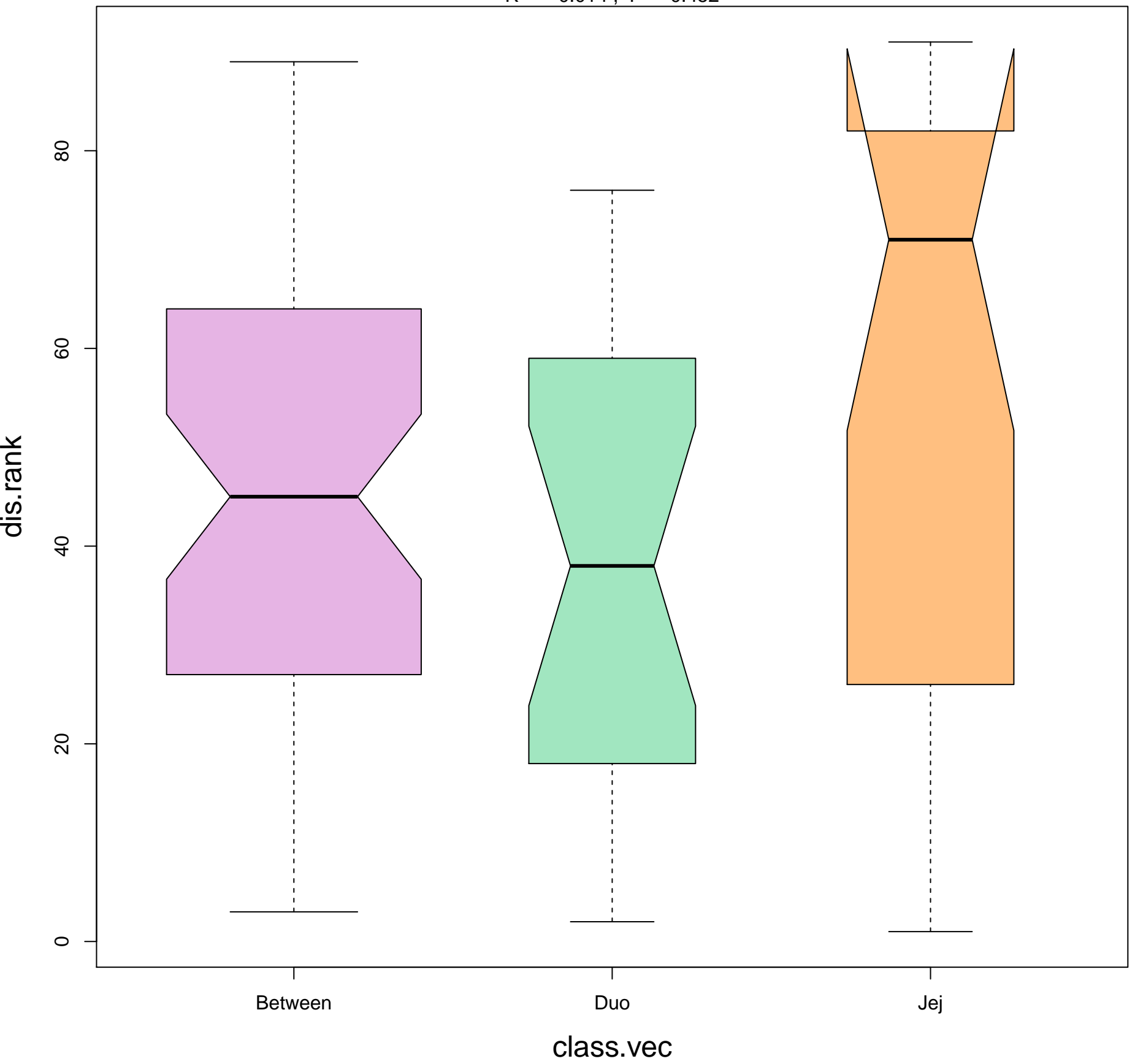

Supplement: Supplementary file 5 [file Data_Sheet_3.zip › Supplementary files 3 ANOSIM similarity analysis results/anosim.Duo_Jej.pdf]

R = 1 , P = 0.001

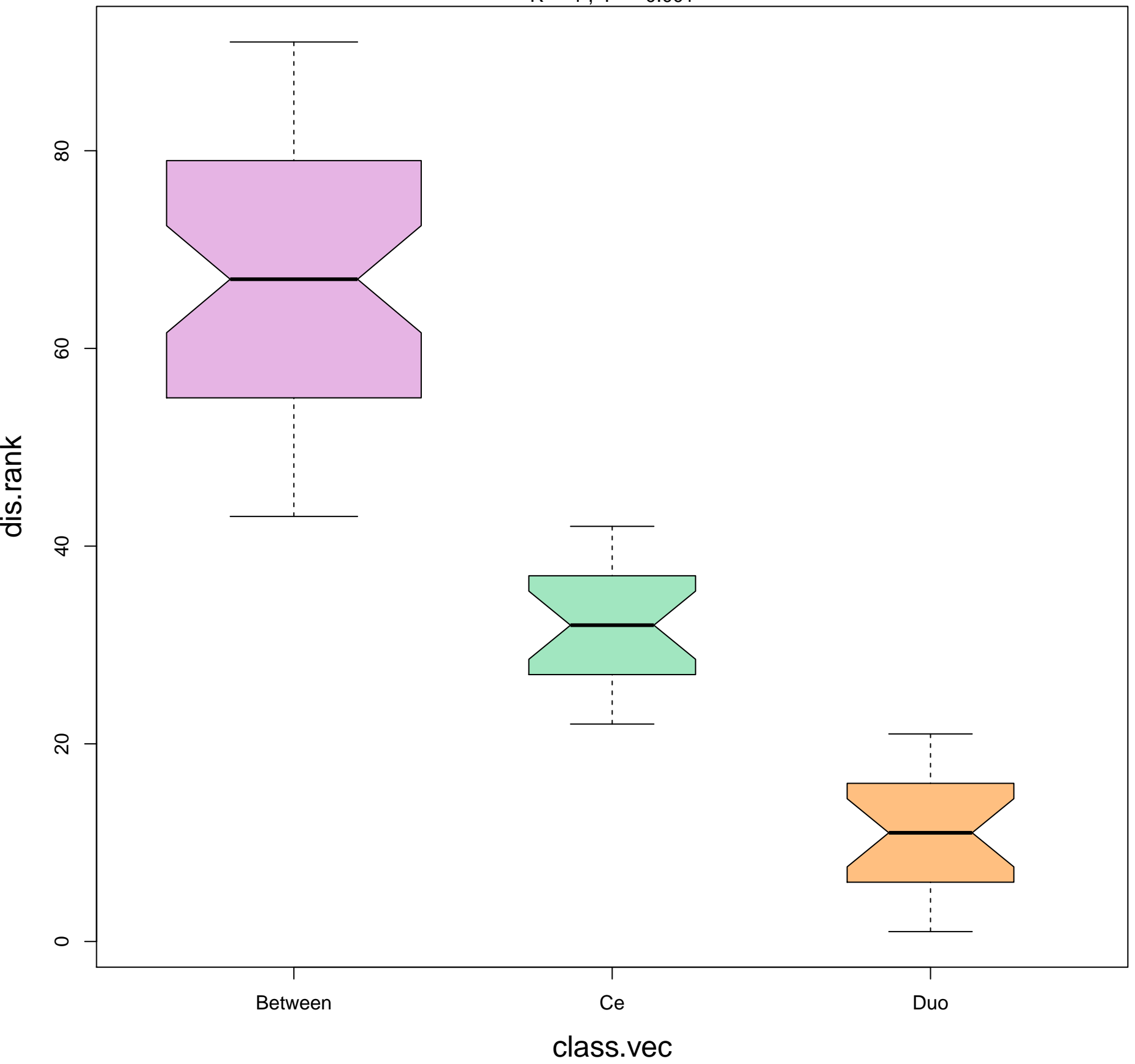

Supplement: Supplementary file 5 [file Data_Sheet_3.zip › Supplementary files 3 ANOSIM similarity analysis results/anosim.Ce_Duo.pdf]

R = 1 , P = 0.001

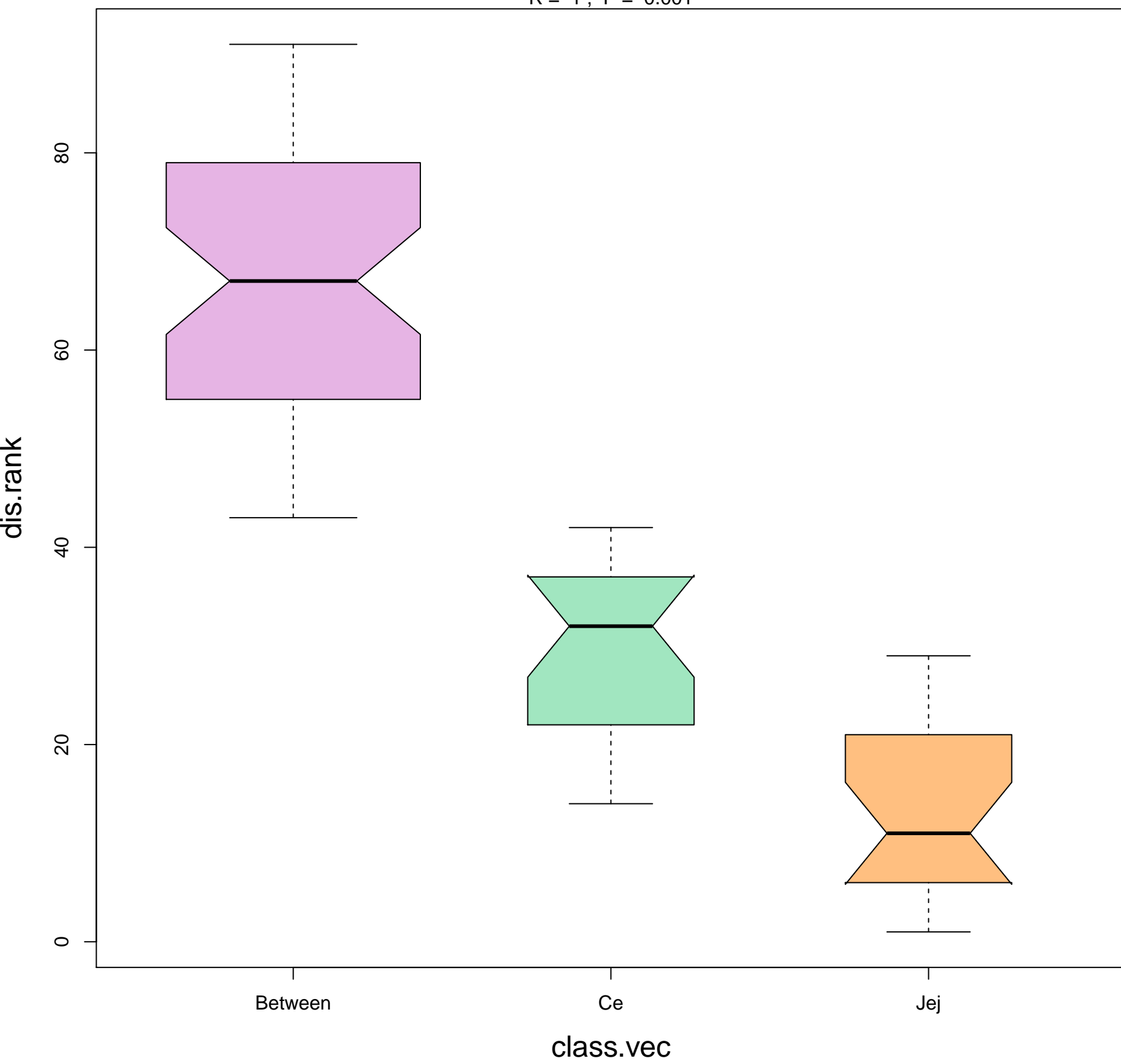

Supplement: Supplementary file 5 [file Data_Sheet_3.zip › Supplementary files 3 ANOSIM similarity analysis results/anosim.Ce_Jej.pdf]

R = 1 , P = 0.001

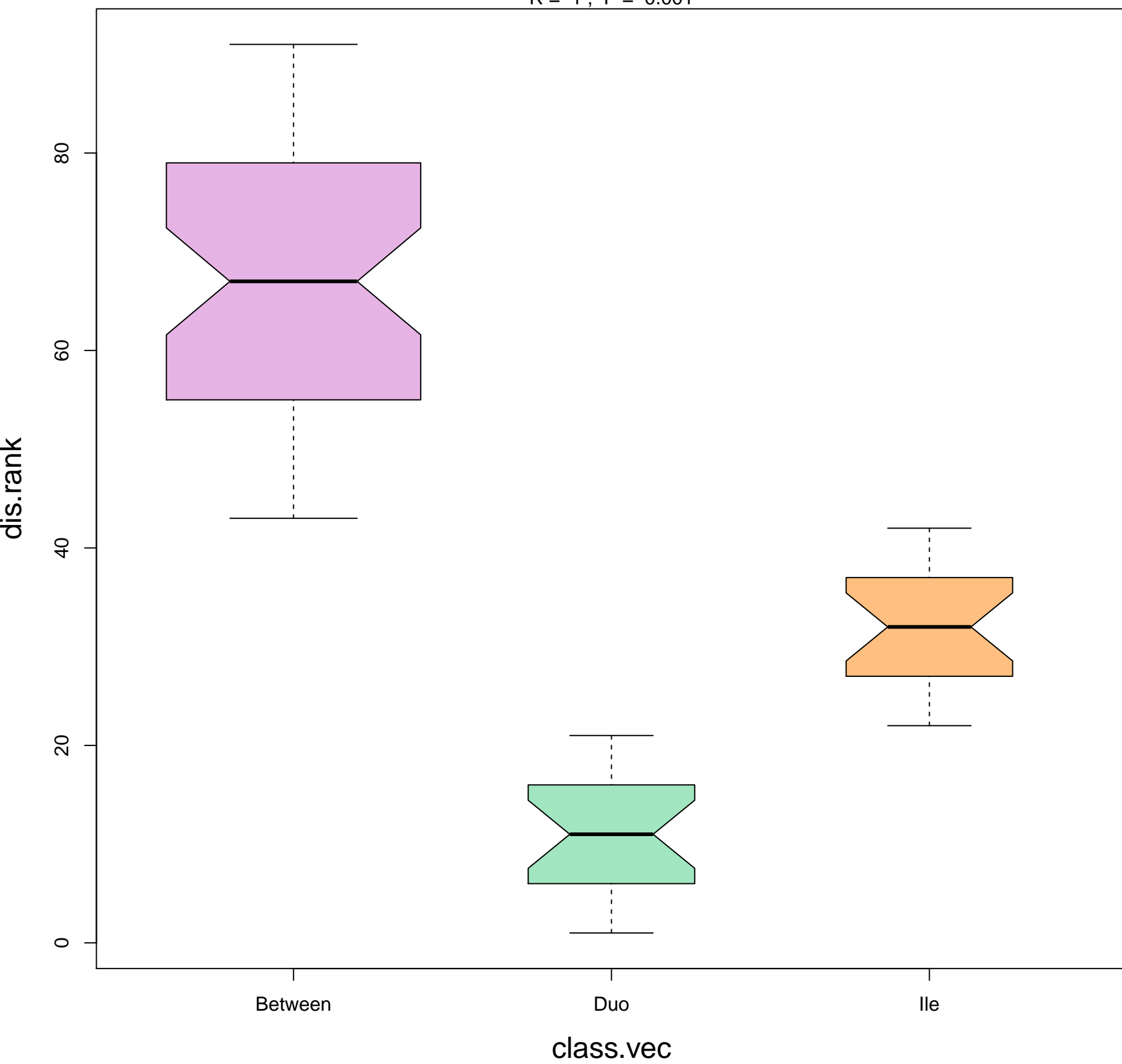

Supplement: Supplementary file 5 [file Data_Sheet_3.zip › Supplementary files 3 ANOSIM similarity analysis results/anosim.Duo_Ile.pdf]

R = -0.145 , P = 0.974

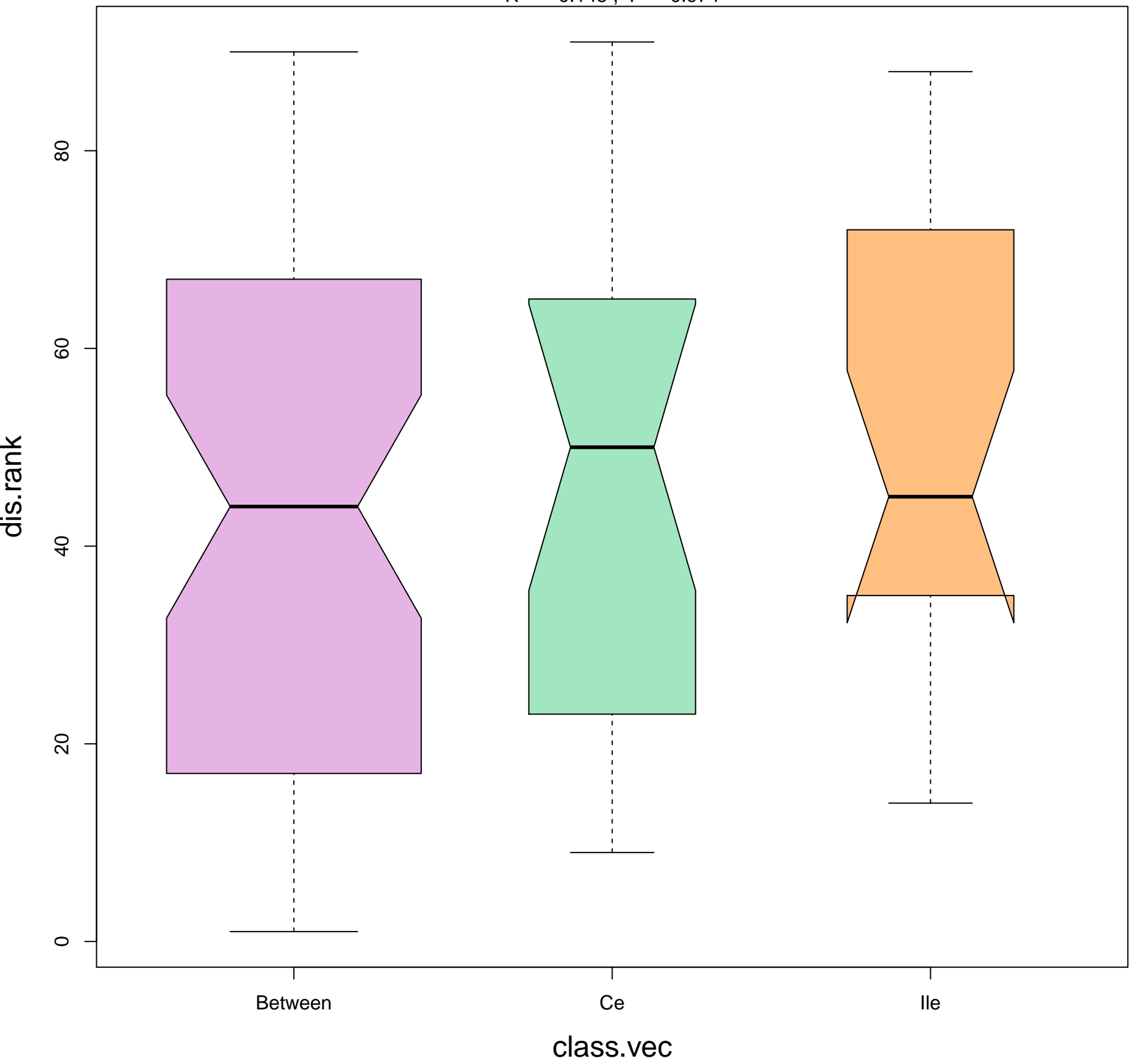

Supplement: Supplementary file 5 [file Data_Sheet_3.zip › Supplementary files 3 ANOSIM similarity analysis results/anosim.Ce_Ile.pdf]

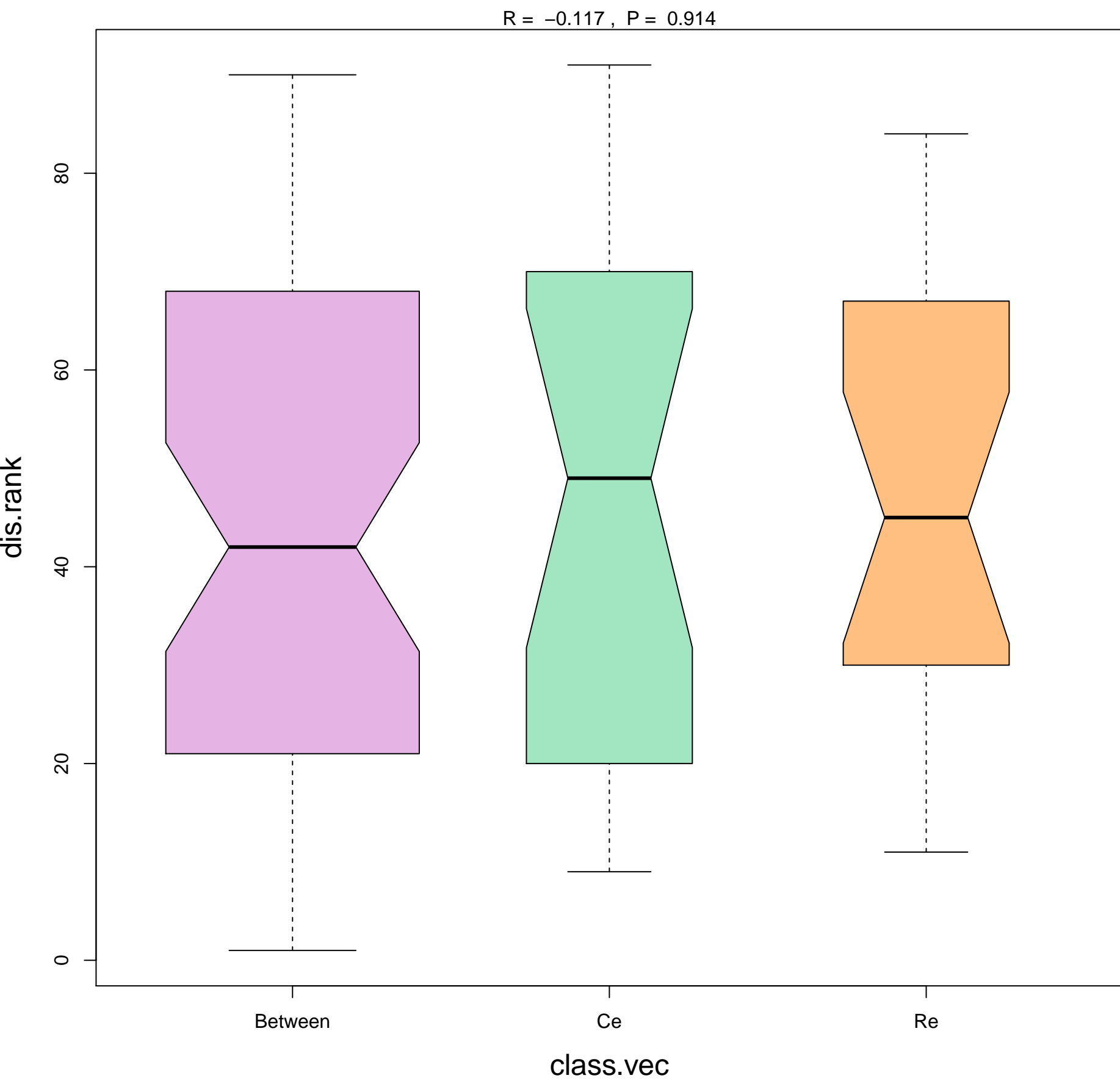

Supplement: Supplementary file 5 [file Data_Sheet_3.zip › Supplementary files 3 ANOSIM similarity analysis results/anosim.Ce_Re.pdf]

R = -0.155 , P = 0.993

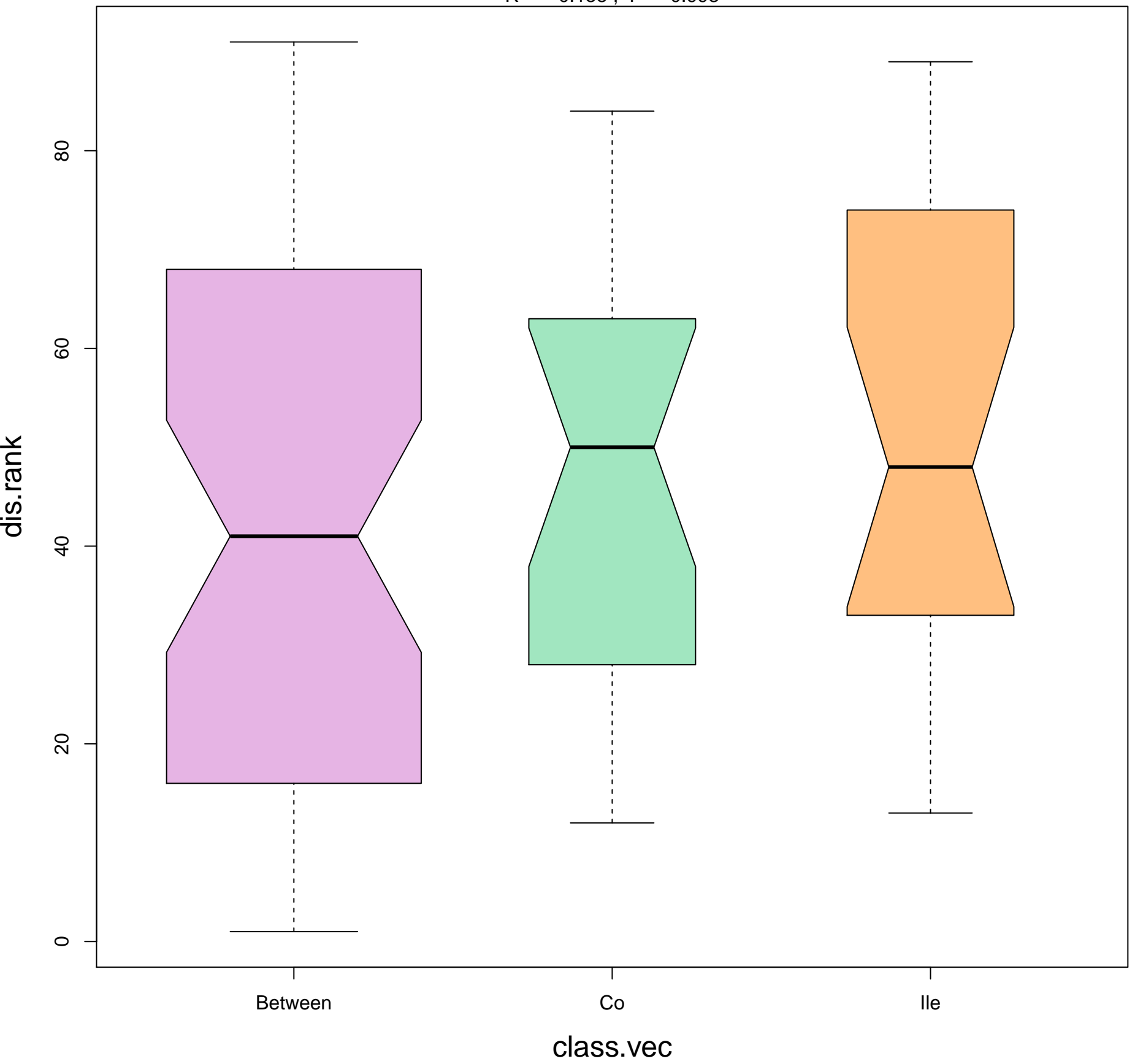

Supplement: Supplementary file 5 [file Data_Sheet_3.zip › Supplementary files 3 ANOSIM similarity analysis results/anosim.Co_Ile.pdf]

R = -0.131 , P = 0.944

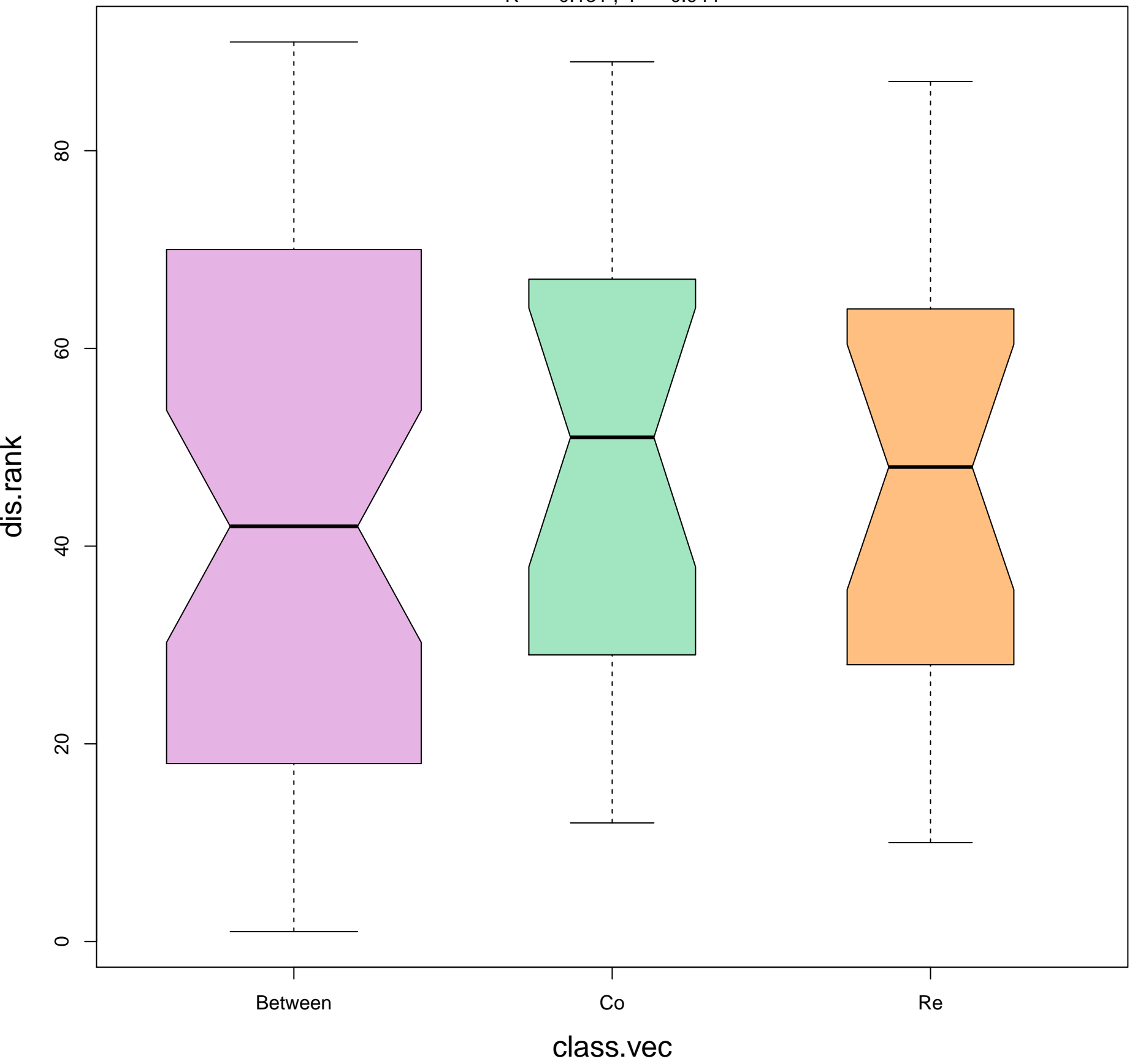

Supplement: Supplementary file 5 [file Data_Sheet_3.zip › Supplementary files 3 ANOSIM similarity analysis results/anosim.Co_Re.pdf]

R = -0.154 , P = 0.996

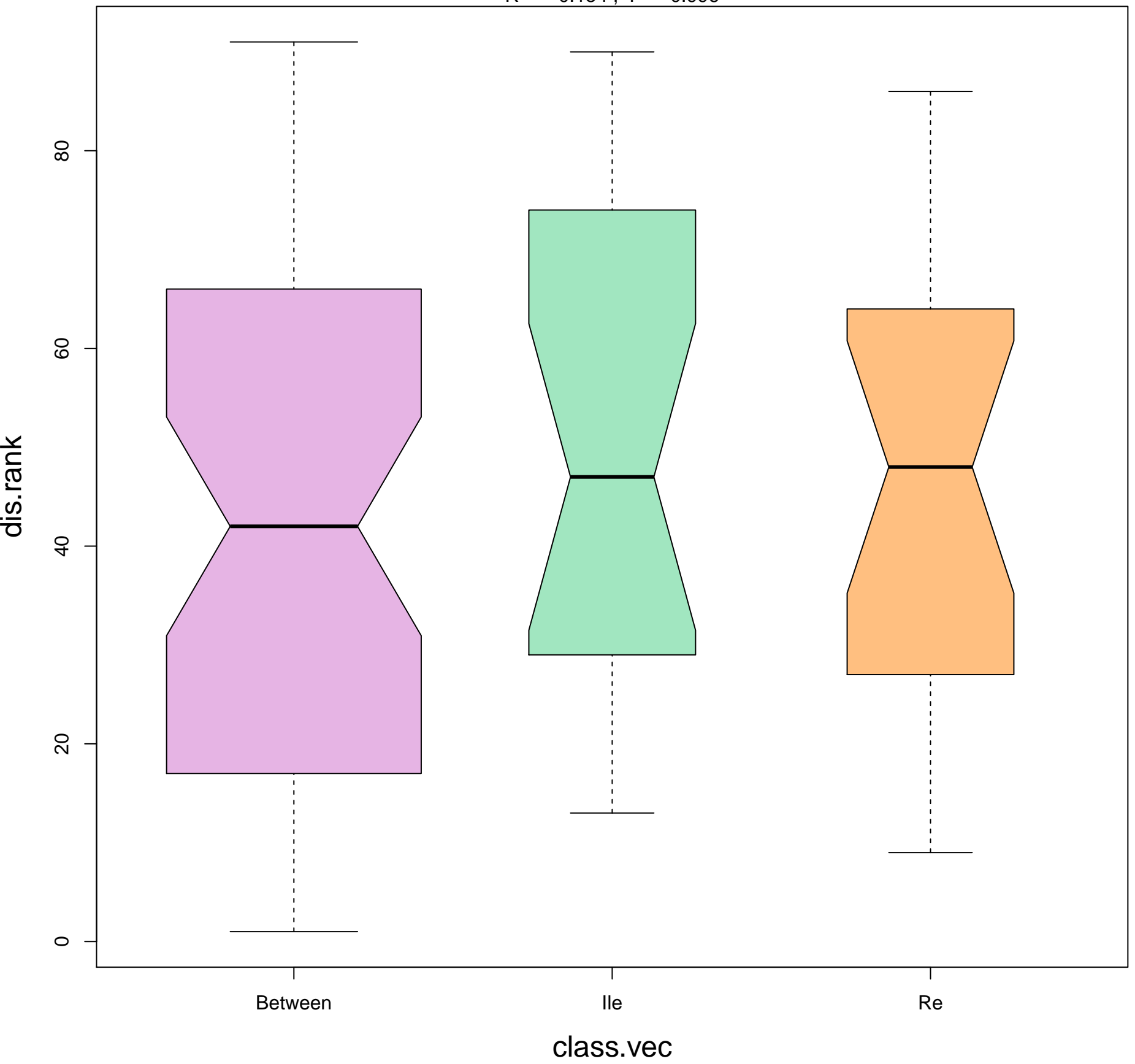

Supplement: Supplementary file 5 [file Data_Sheet_3.zip › Supplementary files 3 ANOSIM similarity analysis results/anosim.Ile_Re.pdf]

R = 1 , P = 0.002

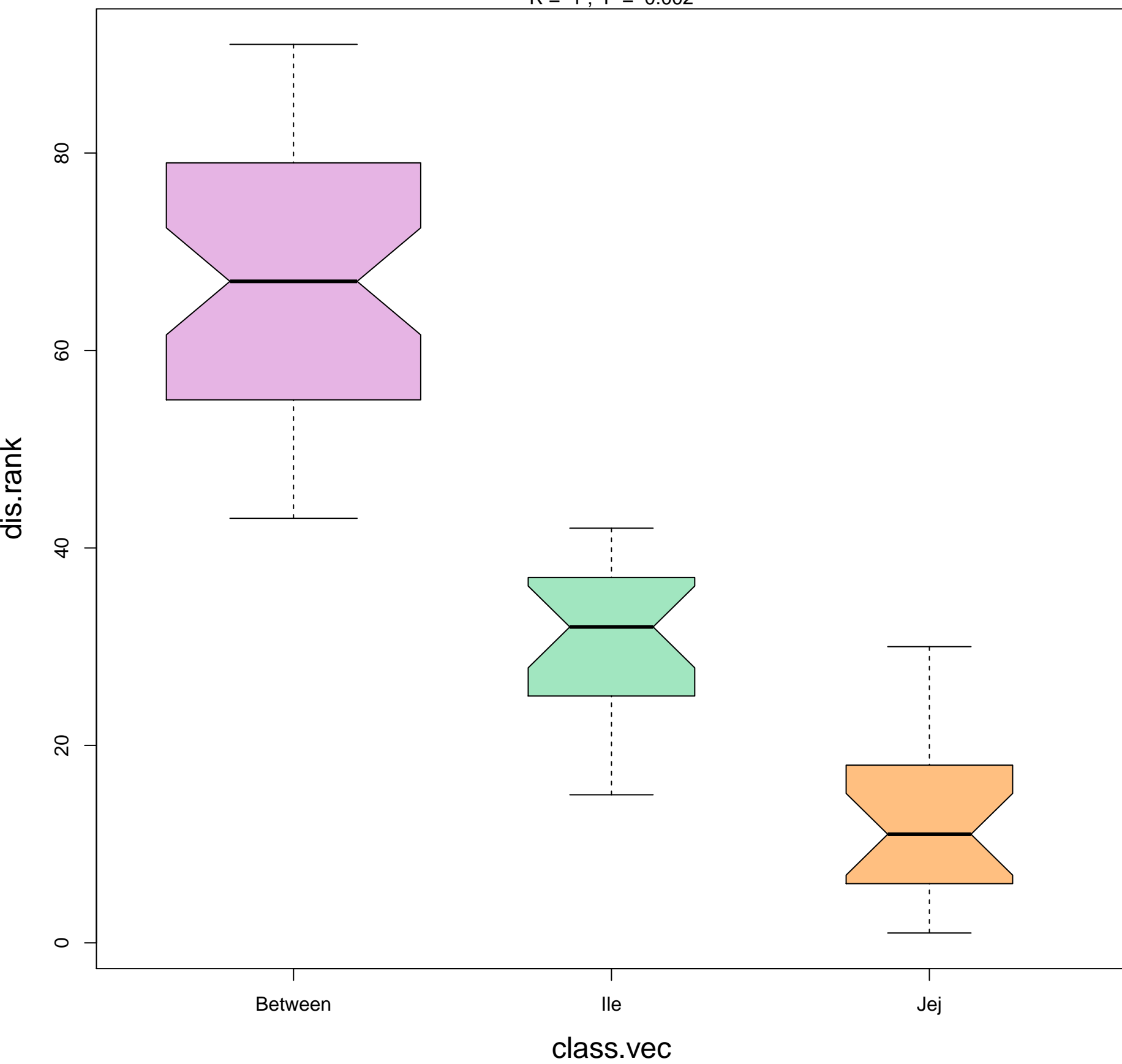

Supplement: Supplementary file 5 [file Data_Sheet_3.zip › Supplementary files 3 ANOSIM similarity analysis results/anosim.Ile_Jej.pdf]

R = 1 , P = 0.002

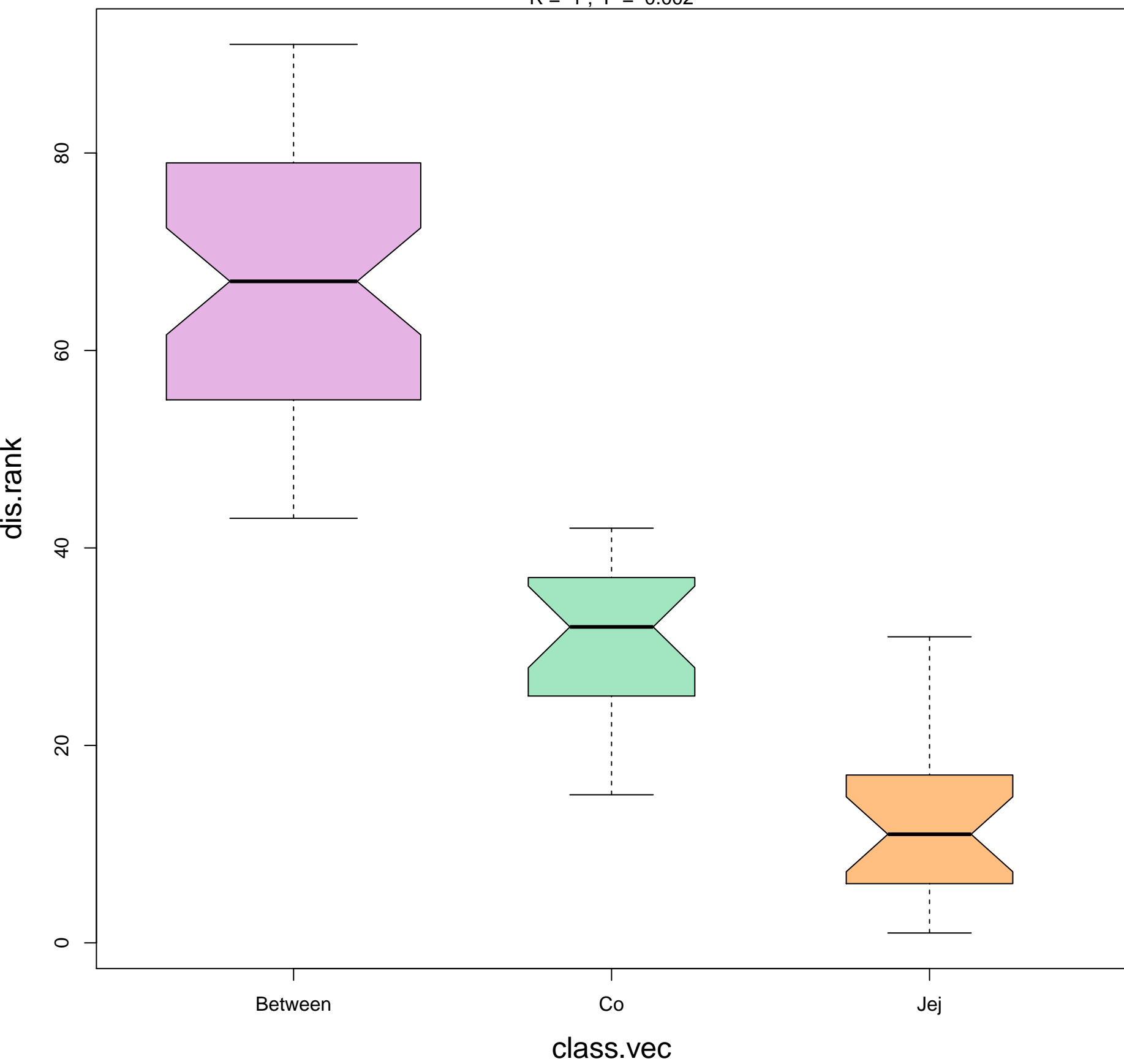

Supplement: Supplementary file 5 [file Data_Sheet_3.zip › Supplementary files 3 ANOSIM similarity analysis results/anosim.Co_Jej.pdf]

R = 0.54 , P = 0.001

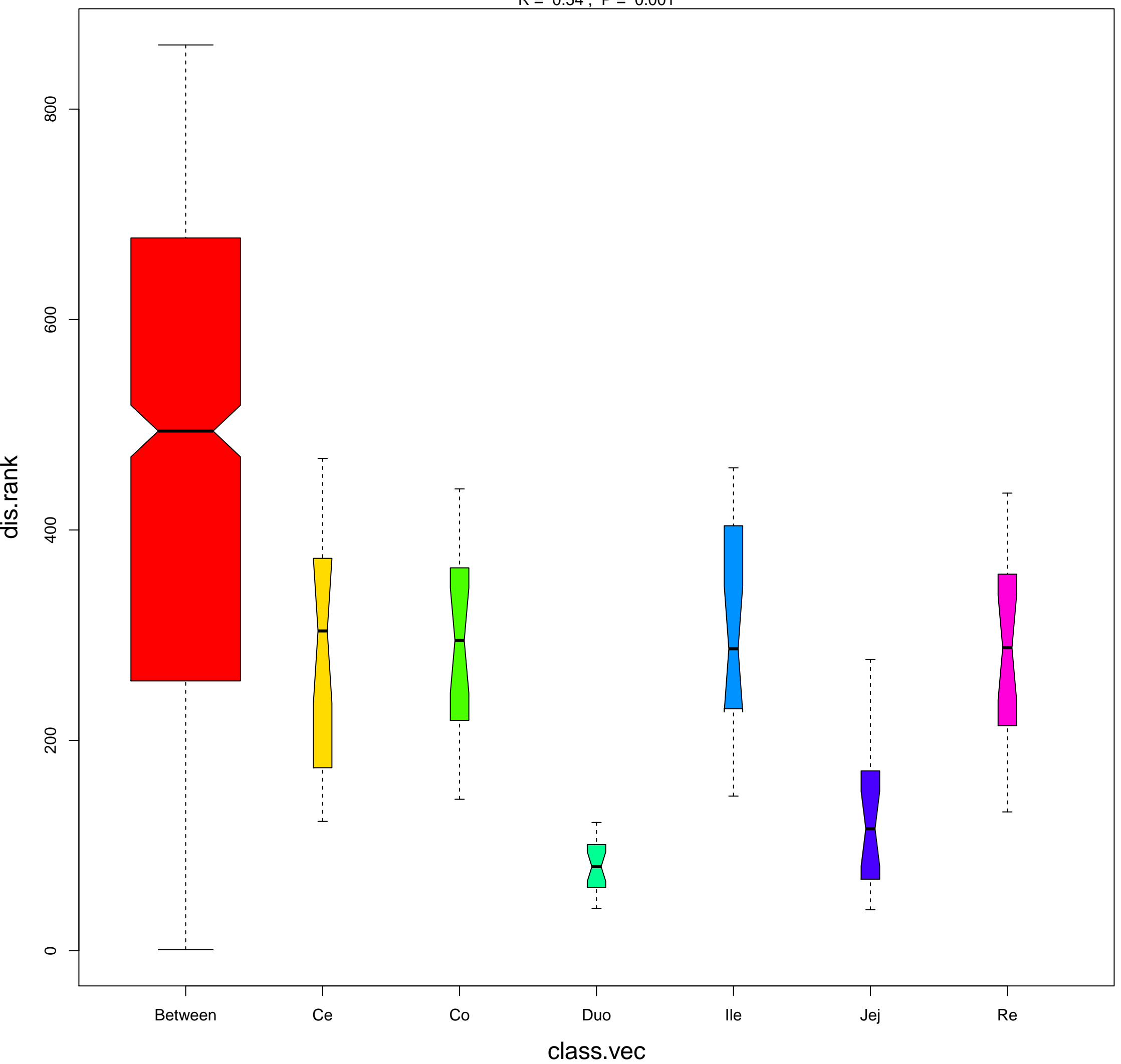

Supplement: Supplementary file 5 [file Data_Sheet_3.zip › Supplementary files 3 ANOSIM similarity analysis results/all group ANOSIM.pdf]

R = 1 , P = 0.001

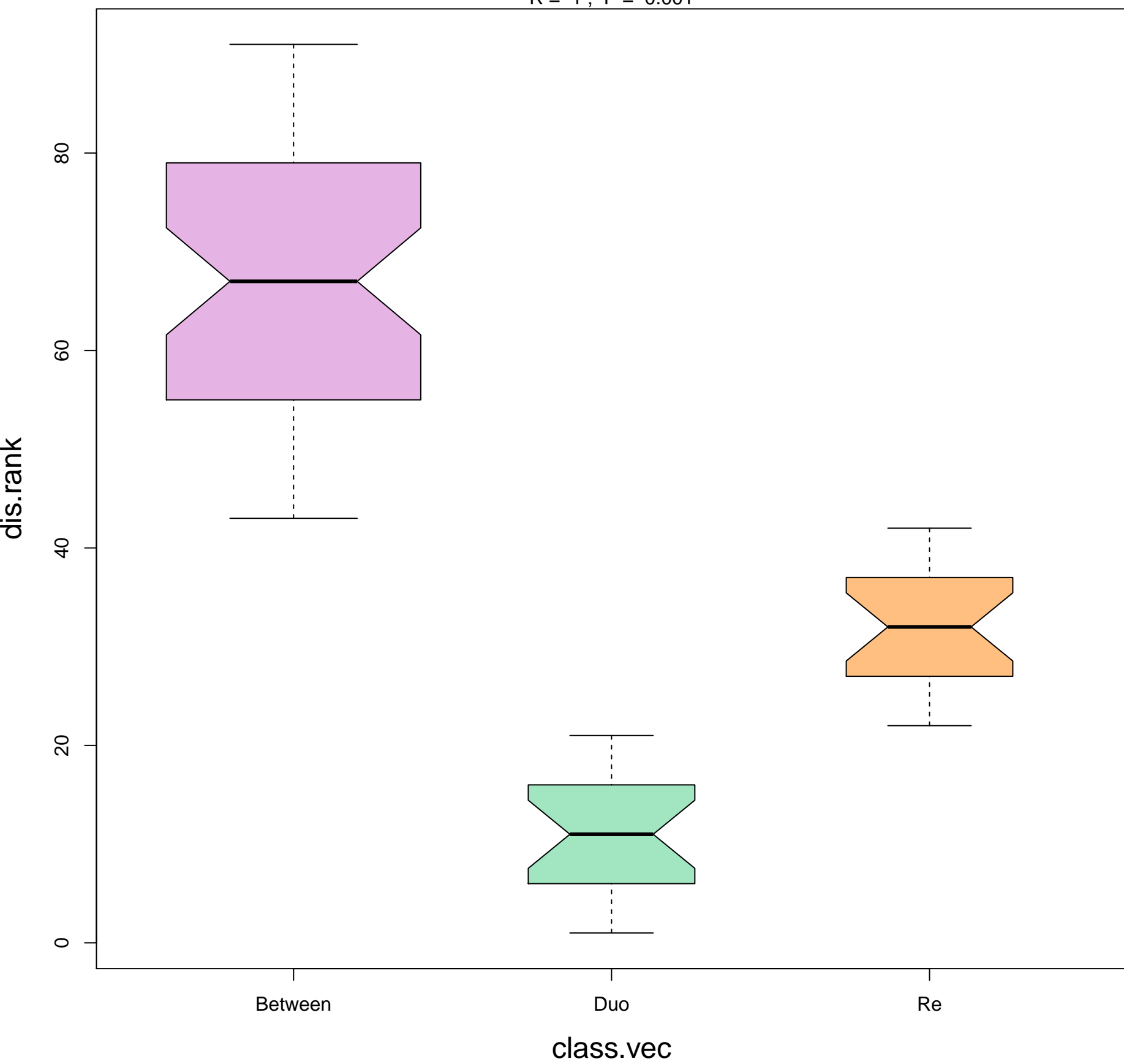

Supplement: Supplementary file 5 [file Data_Sheet_3.zip › Supplementary files 3 ANOSIM similarity analysis results/anosim.Duo_Re.pdf]

R = -0.113 , P = 0.895

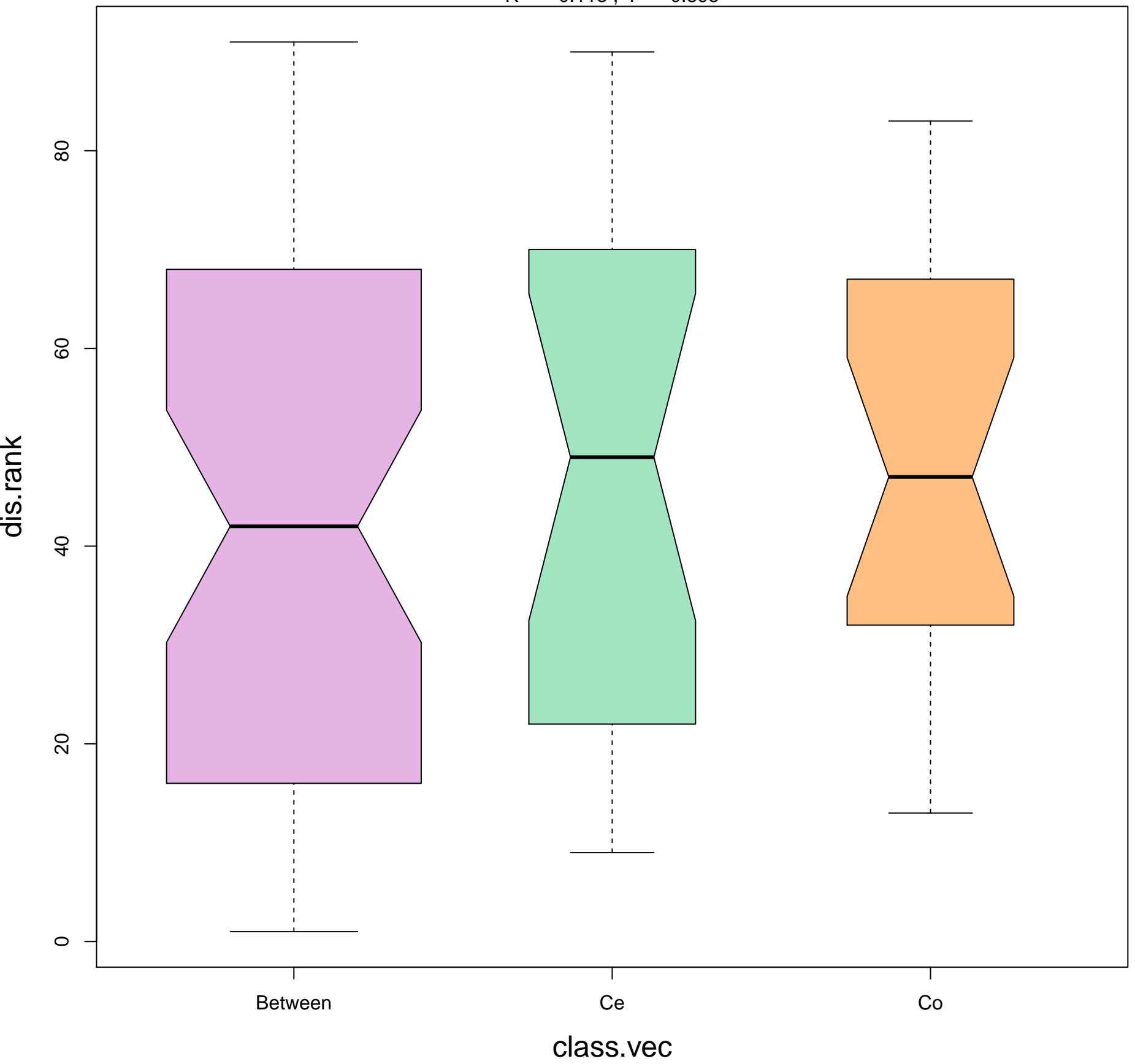

Supplement: Supplementary file 5 [file Data_Sheet_3.zip › Supplementary files 3 ANOSIM similarity analysis results/anosim.Ce_Co.pdf]

R = 1 , P = 0.001

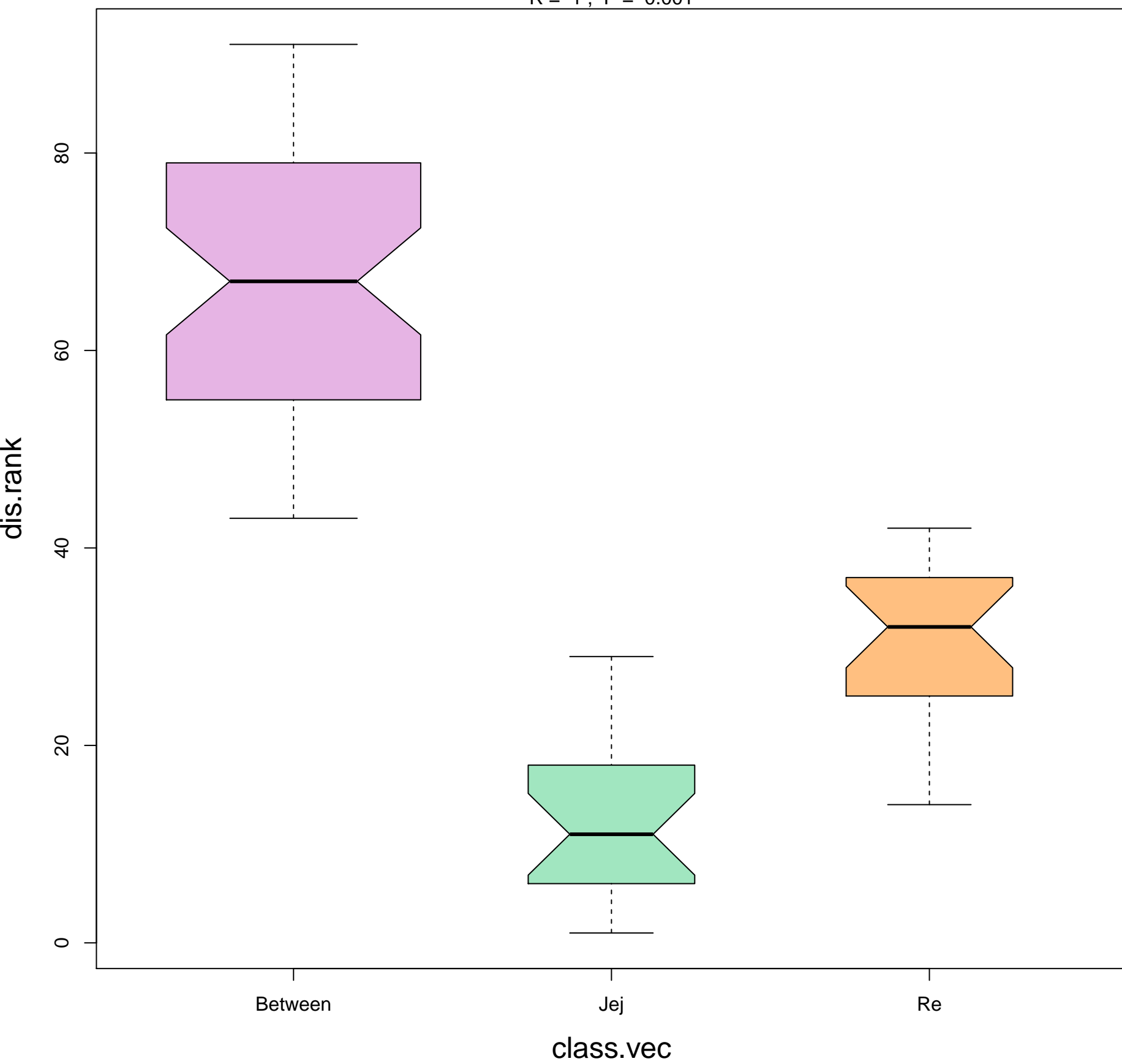

Supplement: Supplementary file 5 [file Data_Sheet_3.zip › Supplementary files 3 ANOSIM similarity analysis results/anosim.Jej_Re.pdf]
